# Comparative Performance Evaluation and Genetic Potential of Frizzle and Deshi Dwarf Chicken Under Intensive Management Condition

**DOI:** 10.1002/vms3.71091

**Published:** 2026-07-15

**Authors:** Bishrat Farhana Amy, Md. Nazmul Haque, Md. Mohan Mia

**Affiliations:** ^1^ Department of Genetics and Animal Breeding Faculty of Veterinary Animal and Biomedical Sciences Sylhet Agricultural University Sylhet Bangladesh

**Keywords:** deshi dwarf chickens, frizzle chicken, intensive management, production, reproduction

## Abstract

**Objectives:**

The objectives of this study were to evaluate and compare the phenotypic and both production and reproduction performance of two indigenous chicken genotypes—frizzle and deshi dwarf—reared under intensive management system. The aim of this study was to identify the superior genotypes which are suitable for intensive management rearing system.

**Methods:**

This work was carried out to compare the morphometric characteristics, production and reproductive performance between frizzle and deshi dwarf chickens under intensive management conditions at Sylhet Agricultural University. A total of 62 chickens, 32 frizzle and 30 deshi dwarf chickens, were monitored. Statistical analyses (SAS) were performed to compare the performance between genotypes.

**Results:**

The mean of beak length, chest girth, keel length, wing length, body length and shank length of frizzle and deshi dwarf were 3.5 ± 0.37 and 2.32 ± 0.38, 26.1 ± 2.8 and 28.55 ± 3.8, 8.26 ± 1.12 and 8.66 ± 0.9, 25.25 + 2.5 and 22.5 ± 2.15, 41.12 ± 4.15 and 30.25 ± 2.6, 6.3 ± 1.03 and 4.75 ± 1.04 cm, respectively. The mean live weights of frizzle and deshi dwarf chickens were 965.35 ± 26.45 and 1073.7 ± 20.46 g at 20 weeks of age, respectively. The average mean of the day‐old chick weight and egg weight, age of first laying of frizzle and deshi dwarf chickens were 28.47 ± 0.1 and 30.43 ± 0.16, 39.82 ± 0.12 and 40.60 ± 0.13 g, 23.25 ± 0.21 weeks and 20.95 ± 0.20 weeks, respectively. The average mean of frizzle and deshi dwarf chickens’ fertility, and hatchability were 82% and 91%, 78% and 86%. The weight of hens at first egg laying was also positively correlated with their age of both chickens. The study revealed a high positive correlation (*p* < 0.05) in frizzle chickens between wing length and shank length (0.98) and in deshi dwarf chickens between body length and shank length (0.97). Additionally, strong positive correlations were found between the weights of chickens at 16 and 20 weeks, with correlation values of 0.96 for frizzle and 0.87 for deshi dwarf chickens.

**Conclusion:**

These findings are expected to assist in making decision for improving the efficiency and sustainability of both chickens in intensive management systems. Comparatively, deshi dwarf chickens are more promising breed under intensive rearing system.

## Graphical abstract

This research work compared and evaluated the morphometric character, productive and reproductive performance of frizzle and deshi dwarf chickens, which were reared under intensive management. In this study, results showed that deshi dwarf chickens had higher phenotypic traits, higher body weight, earlier laying age and greater body weight. Overall, deshi dwarf chicken is more suitable and efficient for intensive rearing systems.

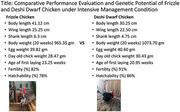

## 1 Introduction

The poultry sector occupies a major portion of the livestock population, and thus, it contributes to the gross domestic product (GDP) of our country. According to Department of Livestock Services (DLS), Bangladesh (2024–2025) (DLS 2024–2025), the livestock contribution to the GDP at current prices was 1.81%. The GDP growth rate of the livestock sector, adjusted for inflation, was 3.19%. The livestock sector accounts for 16.54% of the agricultural GDP at current prices. Bangladesh is home to various indigenous chicken breeds, including Hilly, Naked Neck, Aseel, Yeasine, deshi dwarf, frizzle‐feathered chicken and common deshi birds of non‐idiosyncratic typical type. The indigenous poultry's genetic resources can be fully exploited to improve and diversify breeds for local conditions, benefiting farmers, especially in developing nations (Hinsemu 2018). The commercial strains of chickens and Indigenous family poultry contribute equally to the national egg output at a ratio of 50:50. In terms of meat output, the ratio is 60:40 (Bhuiyan 2011). Indigenous chickens have a predilection for characteristics, such as colour, taste, leanness, hardness, flavour and compatibility with certain cuisines (Hinsemu 2018). They have also good adaptability at adverse environment and produce meat and eggs effectively. Among those, indigenous frizzle chickens appear as if they have gone backward through a windstorm, with their plumage curving outward and forward due to the frizzle mutation affecting the F gene, inherited by incomplete dominance (Dong et al. 2018). They have red eyes, a medium‐sized red single comb, red earlobes, and crimson wattles (Leroy et al. 2018). Frizzle chicken is known for its kind temperament, and its hens are excellent layers that produce medium‐sized eggs in various colours. Additionally, they tend to brood frequently. In contrast, the deshi dwarf chicken, a native breed of Bangladesh, is renowned for its small stature, adaptability to local conditions and efficiency in scavenging systems. This breed has been a cornerstone of rural poultry farming, contributing to the livelihoods of smallholder farmers through egg and meat production (Farooq et al. 2004).

Several studies have highlighted the importance of understanding the morphometric characteristics of poultry breeds as these traits are closely linked to their productive and reproductive performance. Morphometric characteristics, such as body weight, shank length and keel length, are critical indicators of growth potential (Markos et al. 2024). Productive performance also is a crucial determinant of the economic viability of poultry farming. Frizzle and deshi dwarf chickens have been reported to exhibit a moderate growth rate and good feed efficiency under intensive management conditions (Islam et al. 2019; Okeno et al. 2012). Reproductive performance, measured through parameters, such as egg production, hatchability and fertility rates, is another vital aspect influencing the sustainability of poultry breeds. Frizzle and deshi dwarf chickens are known for their consistent egg production and high hatchability rates, making them favourable for commercial egg production. Under intense management conditions, deshi dwarf chickens can outperform native chickens in terms of adult weight and feed consumption without sacrificing egg production (Ferdaus et al. 2016; Dorji and Tamang 2017). This makes them a promising candidate for the intensive management system in Bangladesh, where they could be used to generate smaller layers. This comparative study seeks to provide a comprehensive analysis of the frizzle and deshi dwarf chicken breeds under intensive management conditions, but limited research has yet been conducted in Bangladesh. Therefore, the present study aims to provide a comparative analysis of the morphometric characteristics and productive and reproductive performance of these two breeds under intensive management conditions.

**Objectives**:

- To find out the morphometric traits of frizzle and deshi dwarf chicken.
- To compare the productive and reproductive characteristics of frizzle and deshi dwarf chicken.

## 2 Materials and Methods

**Study area and study period**: The present study was conducted under the department of the Genetics and Animal Breeding Laboratory, Sylhet Agricultural University (SAU) premises, Sylhet, during July 2025–June 2026.

**Selection and collection of birds**: A total of 62 birds were used in this study, including 32 frizzle and 30 deshi dwarf chickens. Birds were collected from different upazilas of Sylhet district, including Golapganj, Sylhet Sadar, Kanaighat and Zakiganj upazila. Birds were transferred to intensive housing conditions at 2 weeks of age.

### 2.1 Management Under Intensive Condition

**Housing and Feeding management system**: The birds were kept in a tin shed house under intensive management. During the experiment, mixed feed was provided to the birds two times, at 8 AM and evening at 5.30 PM. Mixed feed was provided as well as provided fresh water ad libitum. All chickens were vaccinated as per the vaccination schedule. Birds were supplied with commercially prepared grower and layer ration containing approximately 18% crude protein and 2800 kcal ME/kg diet.

**Tagging of sample and egg collection**: To collect accurate data, the chick was tagged by band. The plastic band was put on the leg. The bands are not fixed and do enlarge and are reusable. Eggs were collected two times daily—morning and evening. Eggs were properly marked for record data.

**Incubation and brooding of chicken egg**: After collecting some eggs, they were incubated both naturally and artificially by an electrical incubator. In the electrical incubator, 37.5°C temperature and 60% humidity were maintained during the first week. The relative humidity was raised to approximately 80% during the final 3 days of incubation and artificially brooded by brooder for a period of 3–4 weeks.

**Data collection**: Direct observation was applied during the collection of data. A total of 62 of birds were used for data collection.

### 2.2 Parameters Studied

**Morphometric parameters**: Beak length, chest girth, keel length, wing length, body length and shank length were observed in this study.

**Production parameters**: Live body weight was measured with an electronic balance, from day‐old chick up to mature chicken at 1‐week intervals.

**Reproduction parameters**: Egg weight, age at the first laying of eggs, weight of first laying egg, fertility (%) and hatchability (%) were measured. After getting maturity, birds lay eggs. Eggs were collected daily, marked and stored properly under the supervision of the Department of Genetics and Animal Breeding. Then eggs were incubated in the laboratory of the department. After 21 days, we got the day‐old chicks. All the hicks were tagged and weighted weekly. They were tagged properly so that they could be identified properly. Morphometric characteristics were recorded once a month. At every clutch period we collect eggs and set the incubator and calculate the fertility rate and hatchability rate. Dwarf chickens had a 91% fertility rate and 82% for frizzle chickens. Dwarf chickens generally hatch better, as shown by 86% hatchability rate compared to 78% for frizzle chickens.

**Statistical analysis**: The study's data were coded, put into a Microsoft Excel 2019 worksheet, organized and handled so that they could be used for more research. One‐way analysis of variance (ANOVA) and general linear model (GLM) methods of SAS (1998) software were used to look at the data in a CRD. Duncan's multiple range test (DMRT) was used to see how important the relationships between different traits were. Two genotypes, frizzle and deshi dwarf chickens, were considered two treatment groups. Birds were randomly allocated into three replicates for each genotype. Frizzle chickens (*n* = 32) were distributed into three replicated containing 11, 11 and 10 birds, respectively, whereas deshi dwarf chickens (*n* = 30) were distributed into three replicates with 10 birds per replicate under CRD design.

## 3 Results

**Morphometric traits**: Table 1 presents morphometric traits at 20‐week‐old chicken with significant differences between the two breeds. Frizzle chickens have beaks of 3.5 cm in length, whereas dwarf chickens’ beaks are 2.32 cm in average. The chest girths of frizzle and dwarf chickens are 26.1 and 28.55 cm, respectively. The keel lengths of dwarf chickens and frizzles are 8.66 and 8.26 cm, respectively. Frizzle chickens possess wings of 25.25 cm, whereas dwarf chickens have 22.5 cm. Male individuals from both breeds have higher value compared to their female. The body lengths of frizzle and dwarf chickens are, respectively, 41.12 and 30.25 cm. Frizzle and dwarf chickens possess, respectively, shanks length, respectively, 6.3 and 4.75 cm. These results revealed significant (*p *< 0.05) differences between the two genotypes for most of the morphometric traits.

**TABLE 1:** Morphometric traits of frizzle and deshi dwarf chicken according to sex.

Traits	Sex	Frizzle	Deshi dwarf	LS
Beak length (cm)	Male	3.81^a^ ± 0.036	2.51^b^ ± 0.04	*
	Female	3.17^a^ ± 0.71	2.13^b^ ± 0.72	*
	Overall	3.5^a^ ± 0.37	2.32^b^ ± 0.38	*
Chest girth (cm)	Male	27.25^b^ ± 3.34	30.10^a^ ± 4.37	*
	Female	24.87^b^ ± 2.23	27.00^a^ ± 3.22	*
	Overall	26.1^b^ ± 2.8	28.55^a^ ± 3.8	*
Keel length (cm)	Male	8.75^b^ ± 1.15	9.05^a^ ± 0.97	*
	Female	7.77^b^ ± 1.11	8.27^a^ ± 0.82	*
	Overall	8.26 ± 1.12	8.66 ± 0.9	NS
Wing length (cm)	Male	26.25^a^ ± 3.15	23.25^b^ ± 3.16	*
	Female	24.27^a^ ± 2.12	22.27^b^ ± 1.11	*
	Overall	25.25^a^ ± 2.5	22.5*^b^ ± 2.15	*
Body length (cm)	Male	43.08^a^ ± 4.19	31.25^b^ ± 3.21	*
	Female	39.22^a^ ± 4.14	29.27^b^ ± 2.13	*
	Overall	41.12^a^ ± 4.15	30.25^b^ ± 2.6	*
Shank length (cm)	Male	6.55^a^ ± 1.04	5.10^b^ ± 1.05	*
	Female	5.85^a^ ± 1.02	4.25^b^ ± 1.03	*
	Overall	6.3^a^ ± 1.03	4.75^b^ ± 1.04	*

*Note*: Means with different superscripts along the same column are significantly (*p* < 0.05) different.

Abbreviations: LS, level of significance; NS, non‐significant.

* Indicates statistical significance at *p* < 0.05.

### 3.1 Phenotypic Correlation Among Morphometric Traits

The phenotypic correlation among different morphometric traits (beak length, chest girth, keel length, wing length, wing span, body length, shank length and thigh length) of frizzle and deshi dwarf chicken is presented, respectively, in Tables 2 and 3.

**TABLE 2:** The correlation between different morphometric traits of frizzle chicken.

Traits	Beak length	Chest girth	Keel length	Wing length	Wing span	Body length	Shank length	Thigh length
Beak length								
Chest girth	0.02*							
Keel length	0.018*	0.89*						
Wing length	−0.028*	0.95*	0.94*					
Wing span	−0.038*	0.79*	0.79*	0.87*				
Body length	−0.05*	0.87*	0.76*	0.90*	0.81*			
Shank length	−0.05*	0.94*	0.86*	0.98*	0.87*	0.94*		
Thigh length	0.07*	0.68*	0.56*	0.69*	0.49*	0.68*	0.73*	

* Correlation is significant at the 0.05 level.

**TABLE 3:** The correlation between different morphometric traits of dwarf chicken.

Traits	Beak length	Chest girth	Keel length	Wing length	Wing span	Body length	Shank length	Thigh length
Beak length								
Chest girth	0.84*							
Keel length	0.78*	0.75*						
Wing length	0.89*	0.80*	0.85*					
Wing span	0.79*	0.69*	0.71*	0.84*				
Body length	0.96*	0.85*	0.80*	0.94*	0.84*			
Shank length	0.92*	0.81*	0.71*	0.86*	0.80*	0.97*		
Thigh length	0.89*	0.83*	0.75*	0.86*	0.81*	0.89*	0.87*	

* Correlation is significant at the 0.05 level.

Table 2 displays the correlation coefficients among various morphometric features of frizzle hens, emphasizing some noteworthy associations. Strong positive associations are detected between keel length and shank length (correlation coefficient of 0.98), wing length and wing span (correlation coefficient of 0.95), and chest girth and wing length (correlation coefficient of 0.95). These relationships indicate that when one feature increases, the others are also likely to rise in proportion, indicating interconnected growth patterns in these bodily regions. In addition, there are significant positive correlations between chest girth and keel length (0.89), body length (0.87) and shank length (0.94). This highlights the strong interconnection of these parameters. However, traits that have weaker correlations, such as beak length, may be selected independently depending on various factors. The correlations revealed in this study are statistically significant at the 0.05 level, indicating a strong statistical basis for these relationships.

The phenotypic correlation among different morphometric traits of deshi dwarf chicken is presented in Table 3.

Table 3 shows multiple strong correlations between dwarf chicken morphometric features. Shank, wing and chest girth had the strongest correlations (0.97, 0.89 and 0.96, respectively). Strong positive correlations between these attributes show that as one trait increases, the others increase proportionally. Additionally, chest girth exhibits strong positive associations with beak length (0.84) and shank length (0.92), demonstrating their interconnection. We find some changes in link strength and pattern between these data and frizzle‐chicken correlations. Frizzle chickens have a substantial association between keel and shank length (0.98), although dwarf chickens have a modest correlation (0.75). Frizzle chickens have a greater correlation between chest girth and wing length (0.95) than dwarf chickens (0.69). Dwarf chickens had better associations for body length and chest girth (0.96) than frizzle chickens (0.87).

**Productive traits (live weight)**: Table 4 displays the live weight of frizzle and dwarf chickens categorized by sex and age. These results revealed significant (*p* < 0.05) differences between the two genotypes for most of the productive traits. During the chick stage, both male and female frizzle chickens had a larger average weight (33.20 g for males and 27.86 g for females) compared to dwarf chickens (27.06 g for males and 25.97 g for females). At 20 weeks, the average weight of dwarf chickens (1073.7 g) is higher than that of frizzle chickens (965.35 g), and this difference is statistically significant (*p *< 0.05). Average body weights of frizzle and dwarf chicken was, respectively, 46.00 and 46.12 g at 1 week of age. Higher body weight was found for male bird for both genotypes of chicken (Table 4) at the age of first week.

**TABLE 4:** Live weight (g) of frizzle and dwarf chicken according to sex and age.

Age (week)	Sex	Mean ± SE		LS
		Frizzle	Deshi dwarf	
DOC	Male	33.20^a^ ± 0.30	27.06^b^ ± 0.45	*
	Female	27.86 ± 0.65	25.97 ± 0.32	*
	Overall	30.25^a^ ± 1.12	26.57^b^ ± 1.13	*
1	Male	48.79 ± 2.22	47.71 ± 2.25	NS
	Female	44.63 ± 1.17	44.53 ± 1.18	NS
	Overall	46.00 ± 1.65	46.12 ± 1.71	NS
4	Male Female	155.25^a^ ± 5.78 136.70^b^ ± 4.47	160.70^b^ ± 4.86 145.55^a^ ± 2.48	*
	Overall	145.50^b^ ± 4.8	153.12^a^ ± 3.67	*
8	Male	380.50^b^ ± 12.79	390.30^a^ ± 7.87	*
	Female	346.40^b^ ± 9.94	361.40^a^ ± 6.93	*
	Overall	362.45^b^ ± 11.1	375.77^a^ ± 7.40	*
12	Male Female	595.75^b^ ± 14.07 542.40^b^ ± 16.42	620.50^a^ ± 11.17 574.50^a^ ± 9.43	*
	Overall	523.60^b^ ± 15.20	597.50^a^ ± 10.30	*
16	Male	806.25^b^ ± 29.24	870.90^a^ ± 20.36	*
	Female	726.45^b^ ± 18.78	803.45^a^ ± 16.77	*
	Overall	763.35^b^ ± 24.15	837.17^a^ ± 18.56	*
20	Male	1014.50^b^ ± 31.90	1152.00^a^ ± 22.08	*
	Female	917.10^b^ ± 21.85	995.45^a^ ± 18.84	*
	Overall	965.35^b^ ± 26.45	1073.7^a^ ± 20.46	*

*Note*: Means with different superscripts along the same column are significantly (*p* < 0.05) different.

Abbreviations: LS, level of significance; NS, non‐significant.

* Indicates statistical significance at *p* < 0.05.

Average body weight of frizzle and dwarf chicken was, respectively, 145.50 and 153.12 g at 4 weeks of age. At the age of 8 weeks, average body weight of frizzle and dwarf chicken was, respectively, 362.45 and 375.77 g. At the age of 12 weeks, average body weight of frizzle and dwarf chicken was 523.60 and 597.50 g, respectively. At the age of 16 weeks, average body weight of frizzle and dwarf chicken was, respectively, 763.35 and 837.17 g. At each age point, the weights of males and females in dwarf chickens are consistently higher than those in frizzle chickens (Table 4), suggesting a more vigorous growth pattern.

### 3.2 Phenotypic Correlation Among Productive Traits

The correlation among the live weight of frizzle chicken is presented in Table 5.

**TABLE 5:** Phenotypic correlation between the live weight of frizzle chicken.

Traits	Week 1	Week 4	Week 8	Week 12	Week 16	Week 20
Week 1						
Week 4	0.91*					
Week 8	0.82*	0.84*				
Week 12	0.76	0.65*	0.83*			
Week 16	0.60	0.75	0.79*	0.89*		
Week 20	0.58	0.69	0.74*	0.90*	0.96*	

* Correlation is significant at the 0.05 level.

The highest correlation was 0.96, found among 20‐ and 16‐week ages, which was highly and positively correlated. The correlations between 4 with 1 week, 8 with 4 weeks, 16 with 12 weeks and 20 with 12 weeks were positive and significant, whereas the correlation between 12 with 8 weeks was positive and significant (*p* < 0.05).

The correlation among the live weight of deshi dwarf chicken is presented in Table 6.

**TABLE 6:** Phenotypic correlation between the live weight of deshi dwarf chicken.

Traits	Week 1	Week 4	Week 8	Week 12	Week 16	Week 20
Week 1						
Week 4	0.64*					
Week 8	0.77*	0.65*				
Week 12	0.75	0.76*	0.79*			
Week 16	0.69	0.69	0.74*	0.86*		
Week 20	0.81	0.79	0.73*	0.82*	0.87*	

* Correlation is significant at the 0.05 level.

The table displays the correlation coefficients that measure the strength and direction of correlations between the live weights of hens at various weekly intervals. Consistent growth patterns are evident as the hens age, with significant positive correlations observed throughout most intervals. Weight at Week 1 correlates moderately with Week 4 (0.64) and strongly with consecutive weeks, reaching 0.81 by Week 20. Week 4 correlates moderately with Week 8 (0.65) and stronger with the following weeks, reaching 0.79 with Week 20. All succeeding weeks are correlated with Week 8, especially Weeks 12 (0.79) and 20 (0.73). Week 12 weight is closely associated with Weeks 16 (0.86) and 20 (0.82), demonstrating growth stability. The highest correlation was 0.87, found between Weeks 16 and 20, which was highly and positively correlated.

**Reproductive traits**: The reproductive traits of frizzle and deshi dwarf chicken under an intensive system are presented in Table 7. The egg weights of deshi dwarf and frizzle chickens are, respectively, 40.60 and 39.82 g. The weight of day‐old chicks is greater in dwarf chickens (30.43 g) compared to frizzle chickens (28.47 g). The ages of first egg laying of both dwarf and frizzle chickens are 20.95 and 23.25 weeks, respectively (Table 7).

**TABLE 7:** Reproductive traits of frizzle and deshi dwarf chicken.

Traits	(Mean ± SE)	
	Frizzle	Deshi dwarf
Egg weight (g)	39.82 ± 0.12	40.60 ± 0.13
Age at the first egg laying (week)	23.25^a^ ± 0.21	20.95^b^ ± 0.20
Body weight at first egg laying (g)	1421.40^a^ ± 14.19	1035.50^b^ ± 10.73
Hatchability (%)	78	86
Fertility (%)	82	91

*Note*: Means with different superscripts along the same column are significantly (*p* < 0.05) different.

The weights of first egg laying of both dwarf and frizzle chickens are 1035.50 and 1421.40 g. Dwarf chickens have higher rates of hatchability (86%) and fertility (91%) in comparison to frizzle chickens, which had hatchability and fertility rates of 78% and 82%, respectively. These results revealed significant (*p* < 0.05) differences between the two genotypes for most of the reproductive traits (Table 7).

## 4 Discussion

The present study describes morphometric characteristics, productive and reproductive performance of frizzle‐feathered chicken under an intensive management system and conditions.

### 4.1 Morphometric Traits

**Beak length**: In this study, the average beak length of adult frizzle chickens was found to be 3.84 cm in males and 3.21 cm in females. These measurements are similar to the results reported by Fayeye et al. (2014) and higher than the results of Badubi (2006). In addition, our research reveals that the lengths of our findings are marginally greater than the stated beak lengths of dwarf chickens in Nigeria (Fayeye et al. 2014). This study also found that the lengths of the beaks are slightly longer than the lengths that were reported for deshi chickens in Bangladesh (Azmal et al. 2006). The increased beak length of frizzle chickens perhaps improves their ability to search for food and adapt to different environments.

**Chest girth**: The chest girths of frizzle‐feathered and deshi dwarf hens were 26.1 and 28.55 cm, which is quite similar to investigation of Daikwo et al. (2015). However, it is worth noting that this measurement is slightly smaller compared to the 28.55 cm recorded in dwarf chickens. Tabassum et al. (2014) found that the chest girth of Indigenous chickens in Jhinaigati upazila, Sherpur district of Bangladesh, was 21.92 cm, which is different from our findings. The frizzle‐feathered chicken demonstrated a bigger vertical dimension compared to female chickens in South and Southeast Asia, which is consistent with the findings of our study. The more reduced chest circumference observed in frizzle hens in comparison to dwarf chickens may indicate a more condensed body form, which could potentially impact their respiratory efficiency and overall endurance.

**Keel length**: The study determined that the mean keel length of frizzle‐feathered hens at 20 weeks of age was 9.10 cm for males and 8.28 cm for females, which is smaller than the findings of Bett et al. (2014) and Liyanage et al. (2015). When comparing the lengths of keels in different chicken breeds, frizzle chickens have a length of 8.26 cm, which is similar to dwarf chickens (8.66 cm). However, frizzle chickens have shorter keels compared to the lengths recorded by Daikwo et al. (2015) in Nigeria (12.20 cm) and Ferdaus et al. (2016) for deshi chickens (18.25 cm). Frizzle chickens have keels of sufficient length, but they do not possess exceptional body support and flight skills like other breeds that have longer keels.

**Wing length**: The study results of the wing length of frizzle‐feathered and dwarf hens which is greater than the findings of Daikwo et al. (2015), Birteeb et al. (2016) and Bett et al. (2014). In addition, frizzle chickens exhibited a wing length of 25.25 cm, which was notably longer than the 22.5 cm observed in dwarf chickens. This finding aligns with previous studies conducted by Ferdaus et al. (2016) on deshi hens. It suggests that the longer wings in frizzle chickens are likely advantageous for regulating body temperature and engaging in physical activities.

**Body length**: The study found frizzle‐feathered and dwarf chickens’ body lengths were 41.12 and 30.25 cm long, which are higher than the results of Daikwo et al. (2015), Dorji and Sunar (2014) and Birteeb et al. (2016), but smaller than the findings of Faruque et al. (2015). Ferdaus et al. (2016) reported that Bangladeshi male deshi chickens were 46.23 cm long and females 39.46 cm long. Frizzle chickens may be shorter than deshi chickens. Our study found an increase in body length, which may indicate a higher body mass and egg production potential.

**Shank length**: The study assessed the shank length of frizzle‐feathered and dwarf chickens were, respectively, 6.30 and 4.75 cm, which is similar to the findings of Dorji and Sunar (2014) but smaller than the results of Birteeb et al. (2016) and Daikwo et al. (2015). Faruque et al. (2015) observed that native chickens in Bangladesh raised under intensive systems had a significantly greater average shank length of 9.23 cm, which is higher than our research. Although their ground‐covering capacity may suffer due to the shorter shanks, they may experience fewer leg issues as a consequence of the reduced strain on their legs.

### 4.2 Phenotypic Correlation Among Morphometric Traits

The significant positive relationships among major morphometric traits of both genotypes, such as frizzle and deshi dwarf, are in the present study Tables 2 and 3. The observation can serve as reliable indicators for selection and breeding programmes by using body measurements, especially body length, wing length and shank length, where positive correlation occurred. The moderate to high correlations observed among chest girth, keel length, wing length and thigh length indicate that improvement in one trait may lead to favourable responses in related traits. The significant positive correlations further indicate for faster genetic and developmental influence on these morphometric characteristics. The results demonstrate a high degree of integration among body measurements, supporting the use of correlated traits in selection and conservation strategies for frizzle and deshi dwarf chicken populations.

### 4.3 Productive Traits

The body weights of frizzle and dwarf chickens at different ages and genders reveal their growth trends and meat production capacity. The results confirm the previous study, showing both parallels and variances in chicken breed growth trends. Magothe et al. (2010) in Kenya and Daikwo et al. (2015) in Nigeria found similar beginning body weights of frizzle hens, indicating a similar growth trajectory. Frizzle chickens’ early weights show a continuous rise in body mass, which is typical of this breed. The study found that dwarf chickens outweigh frizzle chicks at all‐time intervals, indicating a stronger development potential. The decreased live weights of frizzle chicks in our study are compared to Bett et al. (2014) and Liyanage et al. (2015). Frizzle hens mature at a lower weight than in South and Southeast Asia, suggesting that environmental, nutritional or genetic factors may affect growth. Faruque et al. (2015) and Ferdaus et al. (2016) provide important information on Bangladeshi native and dwarf chicken development. These data are used to evaluate growth patterns in this study. Due to their higher mature body weight, dwarf chickens are better for meat production due to their improved growth performance (Ferdaus et al. 2016). The significant body weight differences between frizzle and dwarf hens (*p* < 0.05) demonstrate the reliability of these findings. Dwarf chickens are better for meat production than frizzle chickens as they get heavier at each age. In this study, mature body weights of dwarf male and female were, respectively, 1152.00 and 995.45 g. Both breeds show sex‐based weight increase differences. Male chickens weighed more than females, supporting Adomako et al. (2014) findings in Sri Lanka. Male and female growth patterns must be considered when breeding and maintaining animals to enhance meat production. To conclude, dwarf chickens develop faster than frizzle chickens, indicating higher meat output productivity.

### 4.4 Phenotypic Correlation Among Productive Traits

The body weight at early ages may be considered an important selection tool for genetic improvement programmes that enhance growth performance and productivity in indigenous chicken genotypes. The positive phenotypic correlations observed (Tables 5 and 6) in both genotypes, such as frizzle and deshi dwarf, demonstrate that growth traits are closely associated across different ages. The strong relationships indicate that selection for increased body weight at an early age could lead to improvements in body weight at mature age. These findings also support the use of early body weight as a selection criterion in breeding programmes for improving growth performance and productivity in indigenous chicken populations with superior growth potential. The observed results are in agreement with previous studies in indigenous and improved chicken populations, where strong positive correlations between body weights at different ages were reported, indicating that growth traits are genetically and physiologically related.

### 4.5 Reproductive Traits

**Egg weight**: Frizzle chicken acquired in this investigation weighed 38.91 g. Egg weight of 36.30 g of frizzle chicken and 40.06 g at frizzle naked neck reported in Adamawa State were quite similar to the present outcome of the investigation (Bobbo 2013). Faruque et al. (2015) noted that deshi's egg weight of 42.94 g, this outcome was more or less the same. In Sri Lanka, Liyanage et al. (2015) reported that in the intensive system, the mean value of egg weight was 47.11 g for frizzle bird and 48.64 g for naked neck frizzle which was higher than the present result. In Nigeria, Apuno et al. (2011) reported that an egg weight of 39.03 g was closely similar to the result of the present study. Deshi dwarf chickens averaged 40.60 g per egg in this investigation. The average egg weight for dwarf hens in Ferdaus et al. (2016) was 38.25 g, somewhat lower than in our study. However, Faruque et al. (2015) found that native deshi chickens had an average egg weight of 42.94 g, which was higher than dwarf chickens.

**Age at first laying**: This study found that dwarf chickens started laying at 20.95 weeks (Table 7), compared to 23.25 weeks for frizzle chickens. Subalini and Thanuejah (2014) found that frizzle birds averaged 7.26 months and naked neck frizzles 7.09 months at first laying in semi‐intensive systems. Frizzle birds laying at 8.98 months and naked neck frizzle at 8.78 months in intensive systems were older. Adomako et al. (2014) found that adding the frizzle gene to egg‐laying chickens increased egg production and quality, demonstrating that genetics affect laying timing, productivity and quality. Faruque et al. (2015) found that deshi chickens lay at 155 and 147 days, respectively, indicating an early beginning like dwarf chickens in the current study. Frizzle chickens, which start at 23.25 weeks, may mature more slowly due to genetics and phenotypic requirements. Frizzle chickens have improved egg production and quality; however, their somewhat delayed egg laying start may reduce egg yield over time.

**Weight at first laying**: The weight at first laying of frizzle and dwarf chickens were 1421.40 and 1035.50 g, respectively (Table 7). In this study frizzle weight higher than the dwarf chicken. Dwarf chickens have a special character; they can manage body weight and feed intake but they egg produce higher than others indigenous breed. The present study is smaller than the result of Adomako et al. (2014) may be due to environmental effect. The result of Faruque et al. (2015) more or less similar the current study.

**Fertility**: In this study, dwarf chickens had a 91% fertility rate (Table 7) compared to 82% for frizzle chickens. In Nigeria, Adeleke et al. (2012) found 85.7% frizzle‐chicken fertility, demonstrating that given ideal conditions, they can breed well. Regional variances and breeding procedures can affect these results. Similar results surfaced in Nigeria under (Fayeye et al. 2014). The increased reproductive rate of dwarf chickens (91%) in this study is consistent with indigenous chicken breeds in Bangladesh. Faruque et al. (2015) revealed Bangladesh's reproductive rate of Indigenous chicken: 85.4% is considered indigenous that are frizzle‐feathered. According to Bhuiyan (2011), 83% of indigenous chickens in Bangladesh have closely similar fertility with the present result. These studies show that indigenous and dwarf hens are more fertile due to their better environmental adaption and genetic features.

**Hatchability**: Dwarf chickens generally hatch better, as shown by the current study's (Table 7) 86% hatchability rate compared to 78% for frizzle chickens. Subalini and Thanuejah (2014) found that frizzle birds in a semi‐intensive system in Sri Lanka had a hatchability rate of 76.13% and naked neck frizzle birds 79.14%. The intensive method reduced hatchability rates for frizzle birds (72.23%) and naked neck frizzle (71.87%). This illustrates that rearing conditions affect frizzle bird hatchability. In Nigeria, Adeleke et al. (2012) showed a higher hatchability rate of 88.6% for frizzle hens, suggesting regional differences in management, environmental and genetic factors. Indigenous chicken hatchability rates in Bangladesh vary. Indigenous chickens have an 83.8% hatchability rate according to Faruque et al. (2015), and 52% according to Bhuiyan et al. (2013). These variations show that local environmental and managerial factors strongly affect hatchability. Several factors, including genetics, may aid embryo development and survival. According to earlier research, dwarf chickens’ lower maintenance energy needs may improve health and reproductive efficiency, increasing hatchability.

## 5 Conclusion

The study has conducted a thorough analysis of the morphometric features, productivity and reproductive performance of frizzle and deshi dwarf chickens in a controlled intensive management setting. Deshi dwarf chickens showed superior productive and reproductive performance, including higher body weight, earlier sexual maturity, greater fertility and hatchability compared with frizzle chickens. In contrast, frizzle chickens exhibited distinct morphometric characteristics. The findings suggest that deshi dwarf chickens possess better adaptability and production potential under intensive rearing systems and may be considered a promising indigenous genotype for sustainable poultry production in Bangladesh. In summary, this thesis provides significant knowledge regarding the genetic and managerial techniques that can enhance the performance of frizzle and deshi dwarf chickens. The implementation of these findings is expected to assist poultry breeders and farmers in making well‐informed decisions, hence improving the efficiency and sustainability of poultry farming in intensive management systems.

## Author Contributions

**Md. Nazmul Haque**: validation, visualization, writing – review and editing, funding acquisition, investigation, supervision, software, resources, project administration. **Bishrat Farhana Amy**: methodology, conceptualization, writing – original draft, formal analysis, data curation. **Md. Mohan Mia**: investigation, writing – review and editing, supervision.

## Funding

The authors have nothing to report.

## Ethics Statement

The study was conducted with approval (dated 26.10.2025, approval no: 164/25/553) from the Local Ethics Committee for Animal Experiments at Sylhet Agricultural University.

## Conflicts of Interest

The authors declare no conflicts of interest.

## Data Availability

Upon reasonable request, the datasets of this study can be available from the corresponding author.
